# Sodium in relation with nonalcoholic fatty liver disease: A systematic review and meta‐analysis of observational studies

**DOI:** 10.1002/fsn3.2781

**Published:** 2022-02-15

**Authors:** Sara Shojaei‐Zarghani, Ali Reza Safarpour, Mohammad Reza Fattahi, Abbasali Keshtkar

**Affiliations:** ^1^ 48435 Gastroenterohepatology Research Center Shiraz University of Medical Sciences Shiraz Iran; ^2^ 48439 Department of Health Sciences Education Development School of Public Health Tehran University of Medical Sciences Tehran Iran

**Keywords:** nonalcoholic fatty liver disease, sodium chloride, sodium, dietary

## Abstract

Findings on the association of sodium with nonalcoholic fatty liver disease (NAFLD) are conflicting. The present systematic review and meta‐analysis study aimed to assess the association between salt or sodium intake or serum sodium levels and NAFLD risk. Relevant articles were identified by searching PubMed, Web of Knowledge, Scopus, Proquest, and Embase databases through May 1, 2021, without language restriction. The pooled odds ratio (OR) and 95% confidence interval (CI) were estimated using Der‐Simonian and Laird method and random‐effects meta‐analysis. The certainty of the evidence was rated using the GRADE method. Out of 6470 documents, 7 epidemiological/observational (1 cohort, 1 case–control, and 5 cross‐sectional) studies on the relationship between dietary salt/sodium intakes and NAFLD risk met our inclusion criteria. The meta‐analysis of all studies showed a significant positive association between the highest salt/sodium intake and NALFD risk (OR = 1.60, 95% CI: 1.19–2.15) with a meaningful heterogeneity among studies (I^2^ = 96.70%, *p*‐value <.001). The NAFLD risk was greater in the studies with higher quality (OR = 1.81, 95% CI: 1.24–2.65) or using the equation‐based methods for NAFLD ascertainment (OR = 2.02, 95% CI: 1.29–3.17) or urinary sodium collection as a sodium intake assessment (OR = 2.48, 95% CI: 1.52–4.06). The overall certainty of the evidence was very low. In conclusion, high sodium intake seems to be related to increased NAFLD risk. Further well‐designed studies are needed to clarify this association and shed light on the underlying mechanisms.

## Graphical abstract

High sodium intake was related to increased NAFLD risk. The NAFLD risk was greater in the studies with higher quality or using the equation‐based methods for NAFLD ascertainment or urinary sodium collection as a sodium intake assessment. The overall certainty of the evidence was very low.

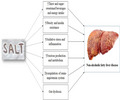

## 1 INTRODUCTION

Nonalcoholic fatty liver disease (NAFLD) is the most prevalent liver disorder affecting 25.24% of the general adult population and 59.67% of diabetic patients worldwide ((Dai et al., 2017); (Younossi et al., 2016)). NAFLD represents excessive intrahepatic fat deposits (>5% of liver weight) in the absence of secondary causes of steatosis, including alcohol abuse, rapid weight loss, total parenteral nutrition, medications, viral infection, or other chronic liver diseases (Kneeman et al., 2012). The pathogenesis of NAFLD involves an imbalance between hepatic fatty acid influx from the diet, adipose tissue lipolysis, and de novo lipogenesis vs. hepatic fatty acid β‐oxidation and very‐low‐density lipoprotein secretion (Nassir et al., 2015). NAFLD is recognized as the liver manifestation of metabolic syndrome and is associated with obesity, diabetes, hypertension, and cardiovascular disease (Wong and Lim, 2018). Along with a genetic predisposition, central obesity, and insulin resistance, dietary factors have critical roles in NAFLD development ((Birjandi et al., 2016); (Dongiovanni et al., 2013); (Nivukoski et al., 2020)).

Salt or sodium chloride is among the most widely used food additives at home and in food industries ((Bansal and Mishra, 2020); (United States Department of Agriculture Aimapp, [internet], 2015)). The global mean salt intake is estimated to be 10.06 g/day (equivalent to 3.95 g/day of sodium) (Powles et al., 2013), which is twice the World Health Organization (WHO) recommendation of <5 g/day (World Health Organization (WHO) Sr, [internet], 2020). High sodium consumption is associated with an elevated risk of obesity (Moosavian et al., 2017), dyslipidemia (Baudrand et al., 2014), hypertension (Subasinghe et al., 2015), cardiovascular disease (Strazzullo et al., 2009), insulin resistance (Baudrand et al., 2014), diabetes (Han et al., 2018), stroke (Strazzullo et al., 2009), and cancer (D’Elia et al., 2012). A high‐salt diet also exacerbated NAFLD and nonalcoholic steatohepatitis (NASH) by inducing inflammation and oxidative stress in rats (Uetake et al., 2015). Besides, a positive association between dietary sodium or salt intake and NAFLD risk, and, subsequently, advice to adherence to a low‐salt diet for NAFLD prevention or suppression in healthy individuals and patients, have been reported in some epidemiological investigations ((Emamat et al., 2021); (Zhou et al., 2021)). Although, some other studies did not confirm these findings ((Portela et al., 2015); (Yang et al., 2015)). No previous article has comprehensively summarized these studies and estimated the pooled effect size so far. Therefore, the present study aimed to conduct a meta‐analysis on observational studies on both genders and all ages and races to seek whether dietary salt or sodium intake or serum sodium levels are associated with the risk of NAFLD.

## 2 METHODS

### 2.1 Search strategy

The preferred reporting items for systematic reviews and meta‐analysis (PRISMA) guideline (Liberati et al., 2009) was followed in the present study. A systematic search in PubMed, Web of Knowledge, Scopus, Proquest, and Embase databases was performed for relevant articles published since inception until May 1, 2021, using the MeSH terms and other related keywords (PubMed search strategy is reported in Table S1). We searched the gray literature using Scopus and Proquest to identify additional documents. The forward and backward citation tracking was also performed for included articles. The protocol of this study is registered in the International Prospective Register of Systematic Reviews (PROSPERO) with the identification number CRD42021232065.

### 2.2 Eligibility criteria and screening methods

Study inclusion and exclusion criteria and research questions were structured using the Population, Exposure, Comparison, Outcome, and Study Design (PECOS) criteria. All original observational articles, including prospective and retrospective cohort, case–control, and cross‐sectional studies (Study Design), in a population of all races and ages and both genders (Population), without language restriction, assessing the association between dietary salt/sodium intake or serum levels of sodium (Exposure), and risk of NAFLD/NASH (Outcome, health condition of interest) were eligible. Studies were included if the comparator was healthy participants (not NAFLD cases) or patients who suffered from low‐grade NAFLD with lower dietary salt/sodium intake (Comparison). The exclusion criteria were in vitro and in vivo investigations, clinical trials, literature reviews, newspapers, books, notes, surveys, letters, editorials, case reports, and clinical trial registration. Moreover, studies on the high‐salt dietary patterns were ineligible. The searching, screening, and selection process were conducted by S.S.Z. and A.R.S. independently. Discrepancies between the two authors were resolved through discussion or by other reviewers.

### 2.3 Data extraction and risk of bias assessment

The two authors independently extracted data, including the last name of the first author, year of publication, country, study design, age, gender, number of participants, dietary assessment tool, sodium intake measurement method, outcome assessment method, follow‐up duration (for cohort studies), levels of salt or sodium, and the number of events in the highest and lowest categories. The data extraction sheet is summarized in Table 1.

**TABLE 1:** Data extraction of epidemiological studies focusing on the association between salt/sodium intake and NAFLD

First author, Year	Population	Design	*N* of subjects (M/F)	Dietary assessment tool	Age (mean (*SD*)/median (interquartile)/range, year)	Sodium intake measurement method	Sodium categories (lowest/highest category)	NAFLD diagnosis method	Outcome
Emamat et al., 2021	Iran	Case–control	999 (430/569) Cases: 196 Controls: 803	FFQ	43.2 (14.1)	Sodium intake (mg/d)	Tertiles (mean: 3183/5143)	Control: Ultrasonography; NAFLD: Fibroscan	Sodium intake was associated with increased prevalence of NAFLD; After subgroup analysis, sodium intake was related to a higher risk of NAFLD only in patients with BMI≥25
Shen et al., 2019	China	Cohort	35,023 (22,629/12,394) (8 years follow‐up)	Self‐reported questionnaire	50.58 (12.33)	Salt intake (g/d)	Three groups (range: <6 g salt (~<2400 mg/d sodium)/≥10g salt (~≥4000 mg/d sodium)	Ultrasonography	High salt intake was associated with a higher risk of NAFLD incidence
Zhou et al., 2021	USA	Cross‐sectional	HSI‐defined NAFLD: 11,022 (5293/5729); FLI‐defined NAFLD: 5320 (2545/2775)	Two 24‐h dietary recalls	51.7 ± 17.6	Sodium intake (mg/d)	Quartile; HSI‐defined NAFLD (median: 2510.5/4258.2), FLI‐defined NAFLD (median: 2520.5/4264.7)	HSI, FLI	Sodium intake was positively associated with NAFLD
van den Berg et al., 2019	Netherlands	Cross‐sectional	6132 (3032/3100)	‐	53.78 (10.21)	24‐h urinary sodium excretion (mmol/day)	Quintiles (mean: 82.14/220.06)	HSI, FLI	Higher sodium intake was positively associated with suspected NAFLD
Choi et al., 2016	Korea	Cross‐sectional	100,177 (46,596/53581)	FFQ	37.2 (32.7–42.1)	Sodium intake (mg/d)	Quintiles; Men (median: 1219/3485), Women (median: 1077/3310)	Ultrasonography, FLI, ALT	Higher sodium intake was related to a greater prevalence of NAFLD
Huh et al., 2015	Korea	Cross‐sectional	27,433 (11,772/15661)	24‐h dietary recalls	51.52 ± 15.71	Estimation of 24‐h urinary sodium excretion by Tanaka's equation (mEq/day)	Tertiles (35.97–127.94/158.26–450.92)	HSI, FLI	High sodium intake was associated with an increased risk of NAFLD and liver fibrosis
Portela et al., 2015	Brazil	Cross‐sectional	229 (58/171)	Three 24‐h dietary recalls	≥60	Sodium intake (g/d)	Yes/no	Ultrasonography	No association was found between sodium intake and NAFLD

Abbreviations: BMI, body mass index; Ca, calcium; CVD, cardiovascular disease; eGFR, estimated glomerular filtration rate; F, female; FFQ, food frequency questionnaire; FLI, fatty liver index; FPG, fasting plasma glucose; HOMA‐IR, homeostatic model assessment of insulin resistance; HSI, hepatic steatosis index; K, potassium; M, male; Mg, magnesium; NAFLD, nonalcoholic fatty liver disease; PUFA, polyunsaturated fatty acids; SFA, saturated fatty acids; TC, total cholesterol; TG, triglyceride; WC, waist circumference.

The risk of bias was assessed by the two authors independently, using the Newcastle–Ottawa Scale (NOS) ((Modesti et al., 2016); (Wells et al., 2000)). Discrepancies between them were resolved through discussion or by other reviewers. The NOS includes three domains, such as selection (point from 0 to 5 for cross‐sectional studies and from 0 to 4 for others), comparability (0–2), and exposure/outcome (0–3). Then, studies were tiered according to the total scores to the following categories: very high (0–3 points), high (4–6 points), and low risk of bias (7–10 points).

### 2.4 Statistical analysis

The primary outcome of the present study was the pooled risk of NAFLD in subjects with high dietary salt or sodium intake or serum sodium levels than the reference category. We also assessed the effects of region, study design, risk of bias, methods of NAFLD ascertainment, and sodium intake measurement tools on the pooled estimates and heterogeneity.

Crude odds ratios (OR) with a 95% confidence interval (CI) were calculated for the included studies. When the data needed to estimate these values were not available in the article, a request for further information was sent to the authors by email. The summary ORs and 95% CI were also estimated for the highest vs. the lowest categories of salt or sodium using the Der‐Simonian and Laird method. A random‐effects model also was selected to compensate for methodological between‐study heterogeneities, that is, population and methodological diversities. Subgroup analysis was performed based on the region, study design, risk of bias, methods of NAFLD ascertainment, and sodium intake measurement tools to assess the source of heterogeneity across studies. Besides, sensitivity analysis was executed to evaluate the impact of removing each investigation on the pooled results. The presence of possible publication bias was not assessed in the current meta‐analysis due to the small number of the included studies (<10) (Tarsilla, 2010). All tests were conducted using Stata MP Version 16 (StataCorp), and *p*‐values <.05 were considered statistically significant.

### 2.5 Certainty of the evidence

The Grading of Recommendations Assessment, Development and Evaluation (GRADE) approach with the ROBINS‐I tool was used to determine the certainty of evidence. This tool grades the evidence as high, moderate, low, or very low strength. Studies can be downgraded according to five domains (risk of bias, inconsistency, indirectness, imprecision, and publication bias) or upgraded based on three criteria (large magnitude of association, dose–response gradient, and residual plausible bias and confounding) (Schünemann et al., 2019). The risk of bias, inconsistency, and indirectness are considered not serious when most information is from low risk of bias studies (most studies are low risk for selected domains of representativeness of the sample and ascertainment of exposure) (Guyatt et al., 2011), I^2^ is <50% (Guyatt et al., 2011), and the study results have generalizability (Guyatt et al., 2011), respectively. Moreover, the imprecision downgrades when there is no statistically significant association, sufficiently narrow 95% CI, or optimal information size (total number of participants >400) or the estimated point or upper or lower bounds of 95% CI is <1.25 (Guyatt et al., 2011).

## 3 RESULTS

### 3.1 Study selection

**FIGURE 1:**
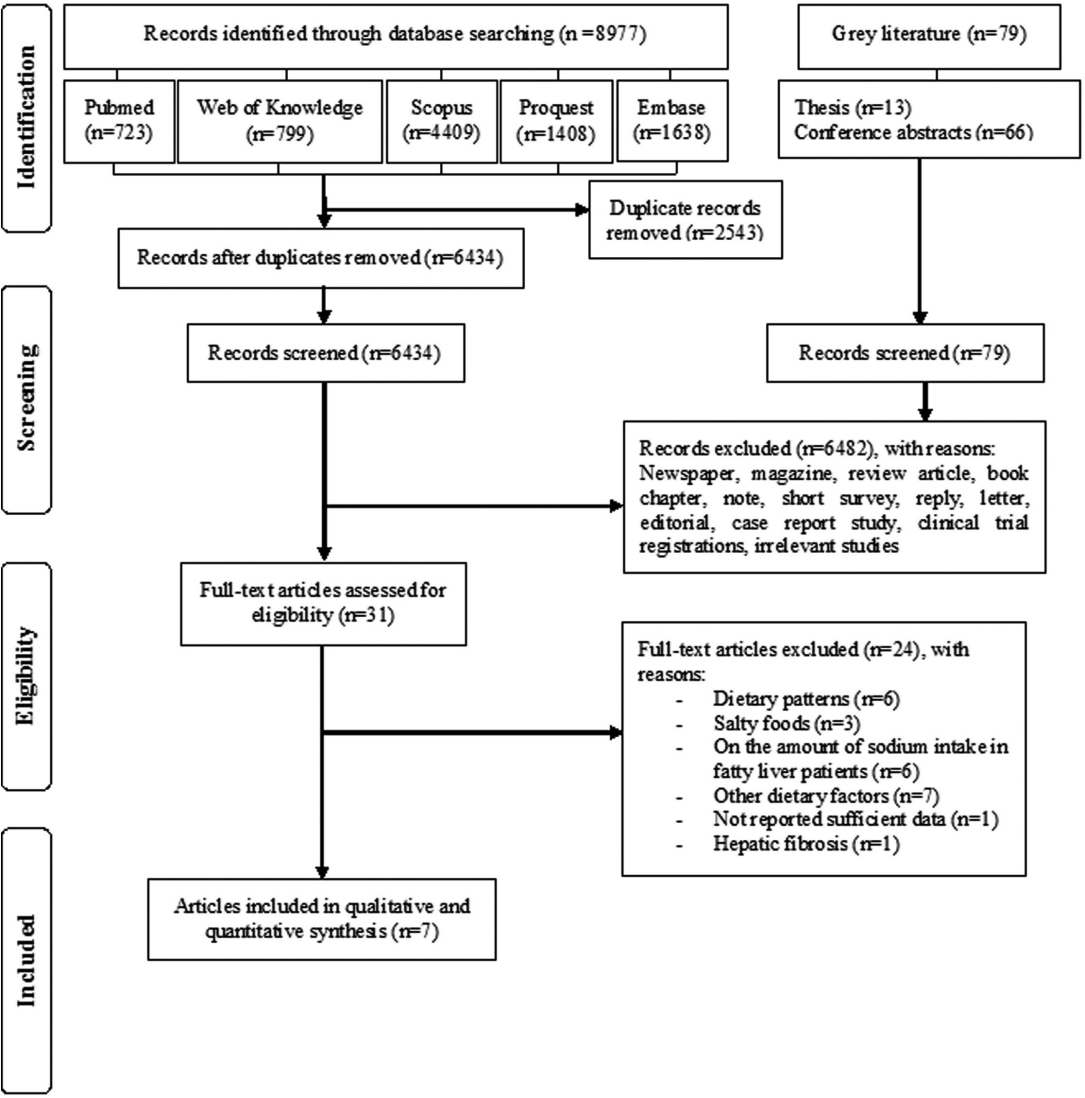
PRISMA flow diagram of the literature search and study selection process

The study flowchart is presented in Figure 1. After the title and abstract screening for 6470 publications, 31 articles were selected for full‐text assessment based on the predefined inclusion and exclusion criteria. Ultimately, seven observational studies published from 2015 to 2020 were eligible for inclusion in the systematic review and meta‐analysis. The list of excluded studies following full‐text assessment is presented in Table S2. No additional articles were found through forwarding and backward citation tracking of eligible studies.

### 3.2 Study characteristics

The main characteristics of the included studies are summarized in Table 1. Of the seven identified articles, one had a prospective cohort design (Shen et al., 2019), one was case–control (Emamat et al., 2021), and the others were cross‐sectional ((Choi et al., 2016); (Huh et al., 2015); (Portela et al., 2015); (van den Berg et al., 2019); (Zhou et al., 2021)). Four studies were done in Asia ((Choi et al., 2016); (Emamat et al., 2021); (Huh et al., 2015); (Shen et al., 2019)), two in America ((Portela et al., 2015); (Zhou et al., 2021)), and one in Europe (van den Berg et al., 2019). All studies were conducted in a population of both genders with an average age of ≥37.2 years old.

Sodium intake was estimated either through measurement of dietary sodium (*n* = 4) ((Choi et al., 2016); (Emamat et al., 2021); (Portela et al., 2015); (Zhou et al., 2021)) and salt (*n* = 1) (Shen et al., 2019) consumption or urinary sodium excretion ((Huh et al., 2015); (van den Berg et al., 2019)). A food frequency questionnaire (FFQ) ((Choi et al., 2016); (Emamat et al., 2021)) or two or three 24‐h dietary recalls ((Portela et al., 2015); (Zhou et al., 2021)) were the used dietary assessment tools in the included studies. Perceived salt ingestion also was assessed through a three‐item questionnaire in one study (Shen et al., 2019). A validated questionnaire was used in all studies, except one (Portela et al., 2015). The mean/median daily sodium intake in the lowest and highest categories were different between studies and ranged from 1077 (Choi et al., 2016) to 3183 (Emamat et al., 2021) and from 3310 (Choi et al., 2016) to 5143 (Emamat et al., 2021) mg, respectively. However, the cutoffs were not reported in one study (Portela et al., 2015).

Nonalcoholic fatty liver disease was diagnosed with ultrasonography in three studies ((Choi et al., 2016); (Portela et al., 2015); (Shen et al., 2019)) and proved with fibroscan in one (Emamat et al., 2021). Fatty liver index (FLI) ≥60 ((Choi et al., 2016); (Huh et al., 2015); (van den Berg et al., 2019); (Zhou et al., 2021)) or hepatic steatosis index (HSI) >36 ((van den Berg et al., 2019); (Zhou et al., 2021)) (or ≥35 (Huh et al., 2015)) also were used as validated markers of suspected NAFLD in four investigations. Due to the better performance of FLI comparing HSI in NAFLD diagnosing, the OR related to the FLI method was included in the meta‐analysis (Chen et al., 2019). The FLI and HSI were calculated according to the following formulas:

FLI=(e^0.953×log^_e_^(triglycerides)+0.139×body mass index (BMI)+0.718×log^_e_^(gamma‐glutamyltransferase(GGT))+0.053×waist circumference–15.745)^/(1+ e^0.953×log^_e_^(triglycerides)+0.139×BMI+0.718×log^_e_^(GGT)+0.053×waist circumference–15.745)^×100 (Bedogni et al., 2006).
HSI=8×(alanine aminotransferase/aspartate aminotransferase ratio)+BMI (+2, if female; +2, if diabetes) (Lee et al., 2010).

### 3.3 Risk of bias assessment

Based on the NOS, the total quality score of the included articles varied from 3 to 8. Therefore, four studies were categorized as "Low," ((Huh et al., 2015); (Shen et al., 2019); (van den Berg et al., 2019); (Zhou et al., 2021)) two as "Very high," ((Emamat et al., 2021); (Portela et al., 2015)) and the other as "High" (Choi et al., 2016) risk of bias. All investigations used a valid method for NAFLD ascertainment, although the technique was not the same for cases and controls in one (Emamat et al., 2021). Random selection of participants was not carried out in two of the included studies ((Emamat et al., 2021); (Portela et al., 2015)), and one study was hospital based (Choi et al., 2016). There was no description of the control selection in the only case–control examination (Emamat et al., 2021). Incomplete control of confounding variables existed in most of the included studies. Exposure was assessed by 24‐h urinary sodium excretion (using two complete consecutive 24‐h urine collections (van den Berg et al., 2019) or Tanaka equation ((Huh et al., 2015); (Huh et al., 2015)) in two studies and by self‐reported questionnaire or unblinded interview for dietary sodium intake estimation in the others (Zhou et al., 2021; Emamat et al., 2021; Portela et al., 2015; Shen et al., 2019; Choi et al., 2016). The validity of the Tanaka formula, which uses spot urine specimens, was weak at the individual level (Zhou et al., 2017). Besides, the response rate was not reported in three articles (Emamat et al., 2021; Portela et al., 2015; Huh et al., 2015) (Table 2).

**TABLE 2:** Detailed results of the risk of bias assessment of the included studies based on the Newcastle–Ottawa Scale (NOS)

Study (Case‐control)	Selection				Comparability	Exposure/outcome			Quality	
	An adequate definition of case	Representativeness of cases	Selection of controls	Definition of controls	Comparability of cases and controls	Ascertainment of exposure	The same method of ascertainment for cases and controls	Nonresponse rate	Total score	Risk of bias
Emamat et al., 2021	✰	‐	‐	✰	✰	‐	‐	‐	3	Very high

Study (cohort)	Ascertainment of exposure	Representativeness of the exposed cohort	Selection of the nonexposed cohort	Ascertainment that outcome was absent at the start	Comparability of cohorts	Assessment of outcome	Adequate follow‐up period	Adequacy of follow‐up of cohorts	Total score	Risk of bias
Shen et al., 2019	‐	✰	✰	✰	✰✰	✰	✰	✰	8	Low

Study (cross‐sectional)	Sample size	Representativeness of the sample	Nonrespondents	Ascertainment of the exposure	Comparability of subjects in different groups	Assessment of outcome	Statistical test		Total score	Risk of bias
Zhou et al., 2021	✰	✰	✰	‐	✰	✰✰	✰		7	Low
van den Berg et al., 2019	✰	✰	✰	✰✰	✰	✰✰	✰		9	Low
Choi et al., 2016	✰	‐	✰	‐	✰	✰✰	✰		6	High
Huh et al., 2015	✰	✰	‐	✰	✰✰	✰✰	✰		8	Low
Portela et al., 2015	‐	‐	‐	‐	‐	✰✰	‐		2	Very high

Note

The total score for the Newcastle–Ottawa Scale is attributed to the following categories: very high risk of bias (0–3 points), high risk of bias (4–6 points), and low risk of bias (7–10 points).

### 3.4 Salt/sodium intake and risk of NAFLD

**FIGURE 2:**
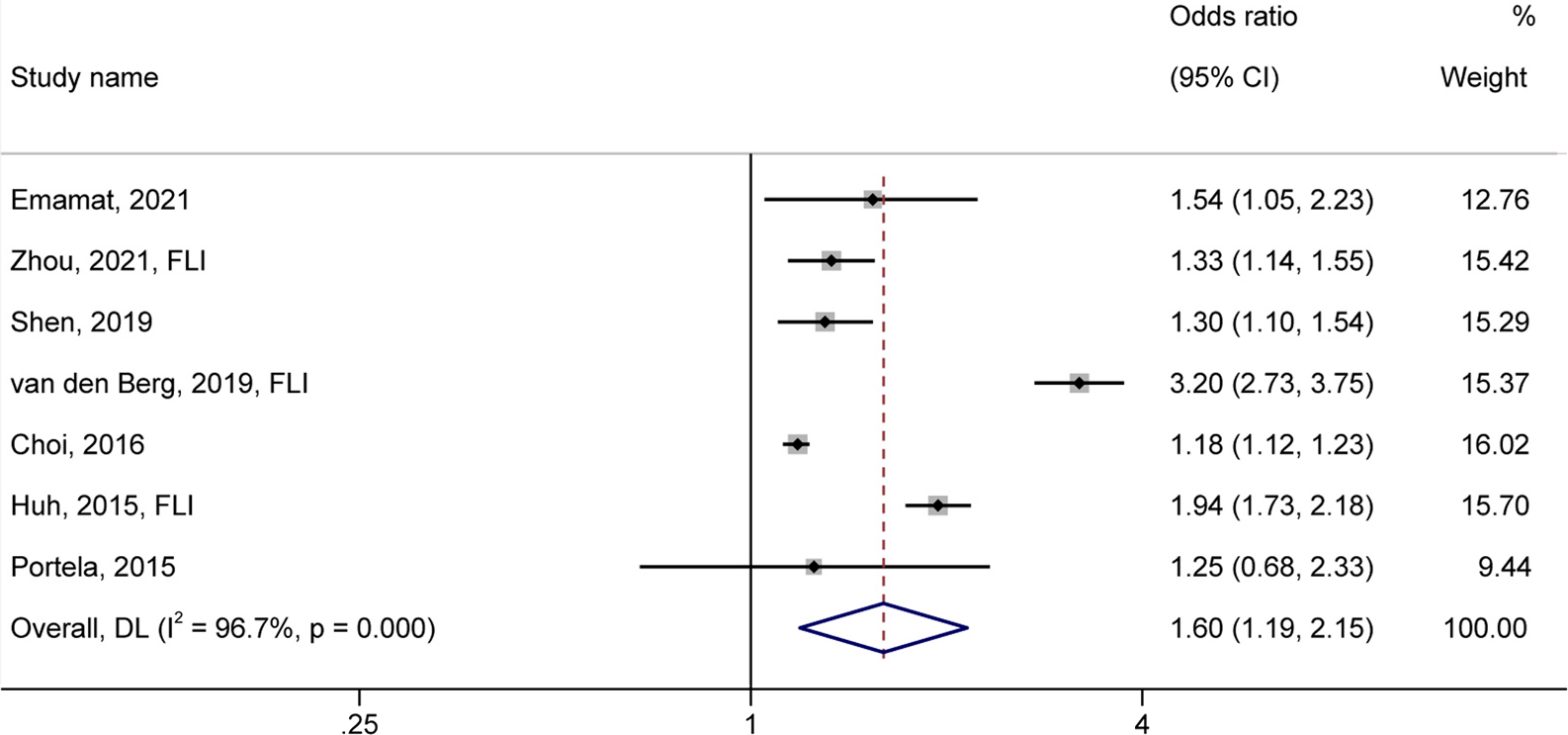
Forest plot (random‐effects model) depicting the association of salt/sodium intake (highest vs.. lowest category) and risk of nonalcoholic fatty liver disease

The result of the meta‐analysis of seven identified epidemiological studies on the association between sodium and NAFLD risk is presented in Figure 2a. The pooled OR was 1.60 (95% CI: 1.19–2.15) for the highest category of sodium as compared to the lowest, with significant heterogeneity among studies (I^2^ = 96.70%, *p*‐value <.001).

**FIGURE 3:**
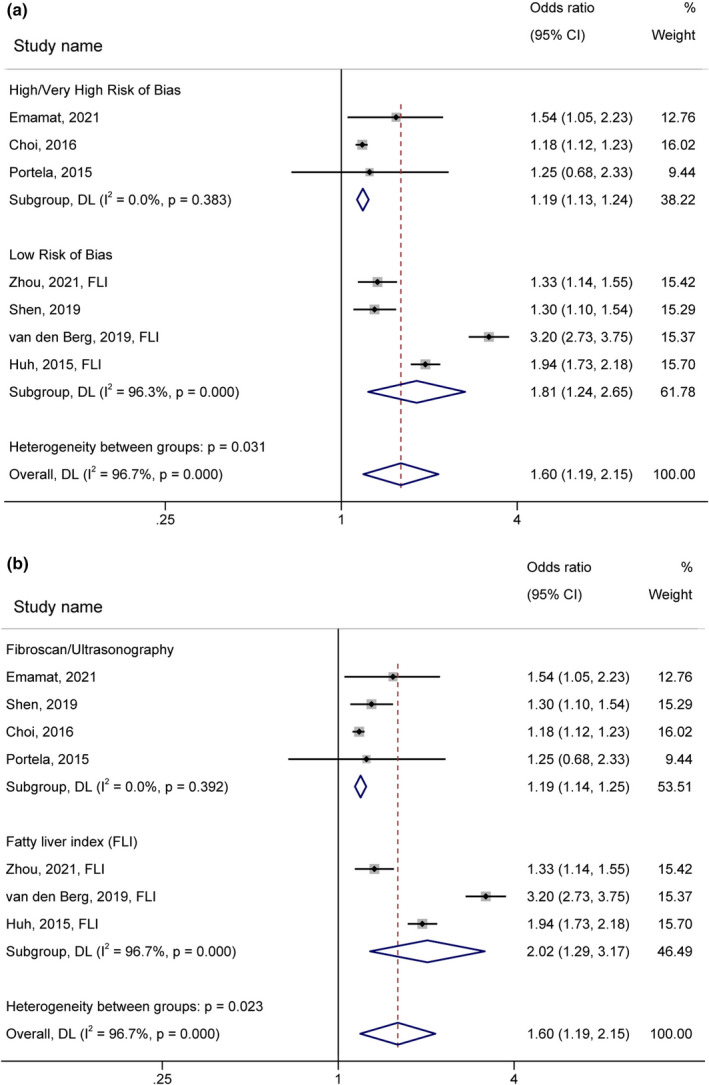
Forest plot (random‐effects model) depicting the association of salt/sodium intake (highest vs.. lowest category) and risk of nonalcoholic fatty liver disease subgrouped by (a) risk of bias and (b) methods of NAFLD ascertainment

**FIGURE 4:**
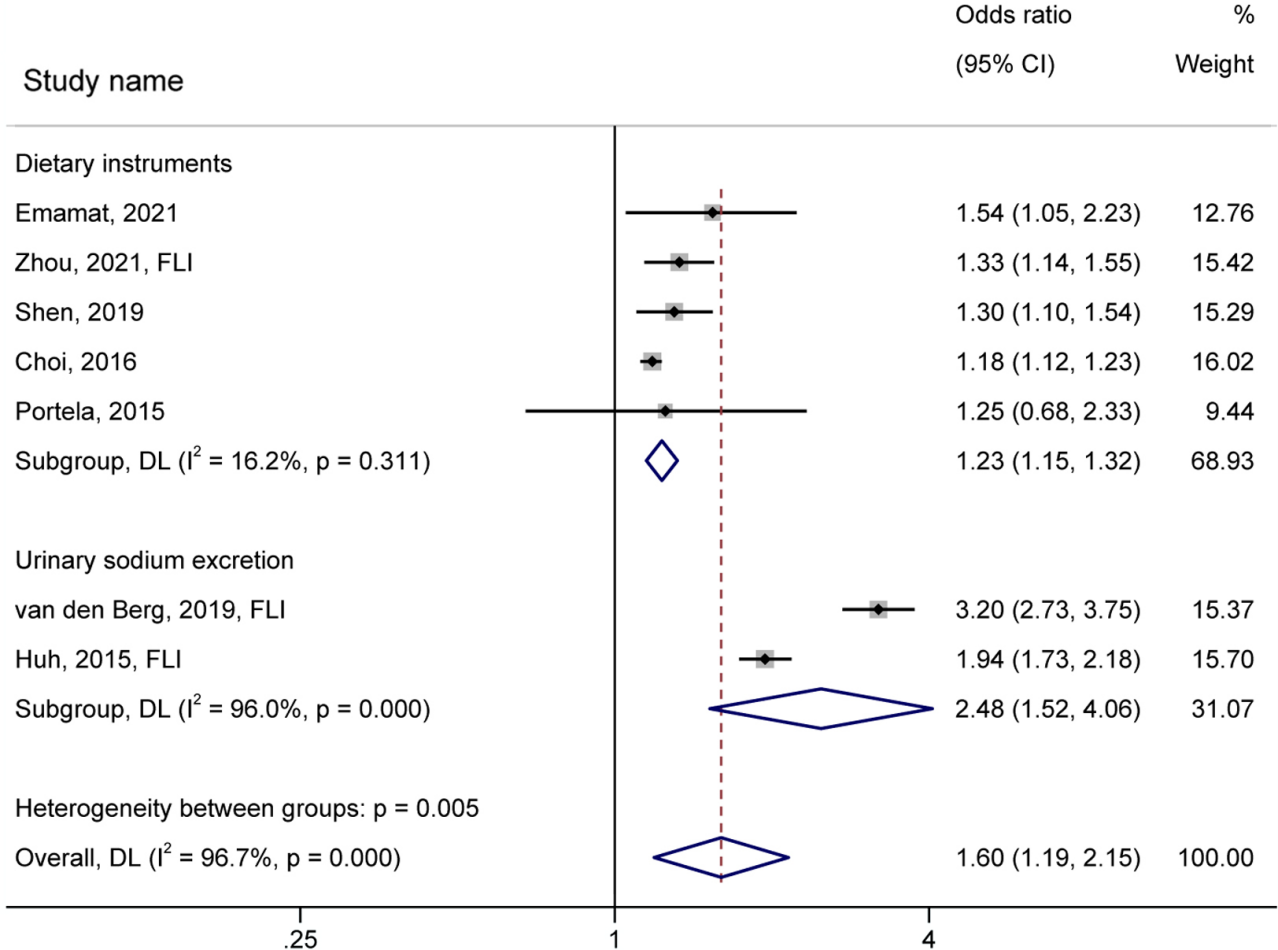
Forest plot (random‐effects model) depicting the association of salt/sodium intake (highest vs.. lowest category) and risk of nonalcoholic fatty liver disease subgrouped by sodium intake measurement tools

The subgroup analysis by region revealed no significant difference between strata (*p*‐value =.597, Figure S1a, OR = 1.46, 95% CI: 1.09–1.95 for studies conducted in Asia and OR = 1.79, 95% CI: 0.88–3.62 for studies performed in America/Europe). There were also no significant differences between cohort/case–control and cross‐sectional studies (*p*‐value =.285, Figure S1b) (Table 3). Stronger association between salt/sodium intake and NAFLD risk was detected in the studies with Low (OR = 1.81, 95% CI: 1.24–2.65, I^2^ = 96.30%) compared to the High/Very high risk of bias (OR = 1.19, 95% CI: 1.13–1.24, I^2^ = 0.00%, *p*‐value =.031, Figure 3a). Besides, a significant greater effect size was observed in the studies using FLI predictive equation (OR = 2.02, 95% CI: 1.29–3.17, I^2^ = 96.70%) than the ones applying fibroscan or ultrasonography diagnostic markers (OR = 1.19, 95% CI: 1.14–1.25, I^2^ = 0.00%, *p*‐value =.023, Figure 3b). Regarding exposure measurement tools, higher urinary sodium excretion (OR = 2.48, 95% CI: 1.52–4.06, I^2^ = 96.00%) was significantly a better predictor of the NAFLD risk compared to the dietary assessment questionnaires (OR = 1.23, 95% CI: 1.15–1.32, I^2^ = 16.20%, *p*‐value =.005, Figure 4).

**TABLE 3:** Stratified meta‐analysis of salt/sodium intake and risk of NAFLD

Characteristics	Number of studies	Summary OR (95% CI)	*p*‐for‐difference	*p*‐for‐heterogeneity	I^2^ (%)
Overall	7	1.60 (1.19–2.15)	‐	<.001	96.74
Region					
Asia	4	1.46 (1.09–1.95)	.597	<.001	95.20
America/Europe	3	1.79 (0.88–3.62)		<.001	96.80
Study design					
Cohort/Case–control	2	1.34 (1.15–1.56)	.285	.421	0.00
Cross‐sectional	5	1.68 (1.14–2.47)		<.001	97.80
Risk of bias					
Low	4	1.81 (1.24–2.65)	.**031**	<.001	96.30
High/Very high	3	1.19 (1.13–1.24)		.383	0.00
Methods of NAFLD ascertainment					
Fibroscan/Ultrasonography	4	1.19 (1.14–1.25)	.**023**	.392	0.00
Fatty liver index (FLI)	3	2.02 (1.29–3.17)		<.001	96.70
Exposure					
Dietary instruments	5	1.23 (1.15–1.32)	.**005**	.31	16.20
Urinary sodium excretion	2	2.48 (1.52–4.06)		<.001	96.00

Abbreviations: CI, confidence interval; NAFLD, nonalcoholic fatty liver disease; OR, odds ratio.

Bold values indicates the significant *p*‐values (*p*<0.05).

#### 3.4.1 Sensitivity analysis

No individual study had a considerable influence on the pooled estimates (Figure S2). The pooled ORs of the sensitivity analysis ranged from 1.70 (95% CI: 1.24–2.33) to 1.41 (95% CI: 1.14–1.75) upon omitting the Choi et al. (Choi et al., 2016) and van den Berg et al. (van den Berg et al., 2019) studies, respectively.

### 3.5 Certainty of the evidence

Table S3 represents the GRADE assessments for the associations between salt/sodium intake and NAFLD risk. The overall certainty of the evidence was very low due to downgrades for risk of bias, indirectness, and imprecision.

## 4 DISCUSSION

As a result of the growing global incidence of NAFLD in recent years, numerous investigations have studied the potential risk factors and intervention strategies for its prevention and management. Excess salt intake is among the major health risks worldwide and a possible factor in the pathogenesis of some hemodynamic and metabolic abnormalities. The main sources of dietary salt appear to be bread and bakery goods, meat and its products, instant noodles, salted preserved foods, dairy products, and condiments (Menyanu et al., 2019; Campbell et al., 2012). To the best of our knowledge, this is the first comprehensive systematic review and meta‐analysis study assessing the relation between salt/sodium intake and risk of NAFLD prevalence or incidence.

The present meta‐analysis indicated a positive association between high salt/sodium consumption and NAFLD risk. The pooled OR indicates a 60% greater risk of NAFLD in high salt/sodium intake than the reference category (*p*‐value <.001) (Figure 2). This association was significantly greater in the studies with higher quality (*p*‐value =.031) or using equation‐based method for NAFLD ascertainment (*p*‐value =.023) or urinary sodium collection as a sodium intake assessment (*p*‐value =.005) (Table 3). Sensitivity analysis revealed that no particular study had a significant influence on the pooled estimates (Figure S2). Due to the observed considerable heterogeneity, because of the differences in genetic backgrounds, age, health condition of participants, study designs, and methods of NAFLD ascertainment and sodium intake assessment, interpretation of findings should be made with caution. Our consequence was in line with previous investigations reporting higher NAFLD risk with adherence to salt‐rich dietary patterns or higher salty snack intake (Choi et al., 2016; Chung et al., 2019; Yari et al., 2020). However, this relation has not been confirmed in all studies (Yang et al., 2015; Alferink et al., 2020). These contradictory results could be partially due to the differences in genetic background, food items included in each dietary pattern, and intake of other nutrients.

Liver biopsy is the gold standard, although invasive, diagnostic tool for NAFLD. This disease usually is screened and diagnosed with blood‐based biomarkers (alanine aminotransferase, aspartate aminotransferase, and GGT), imaging‐based technology (ultrasonography, magnetic resonance imaging, and controlled attenuation parameter), or estimation equations (FLI, HSI, NAFLD liver fat score, and SteatoTest) (National Guideline Centre (UK), NICE Guideline, No. 49., 2016). Besides, there are different methods for evaluating sodium intakes, such as 24‐h urinary collection as the gold standard, spot urine methods, and dietary surveys (24‐h dietary recall, FFQ, and diet record) (Defagó and Perovic, 2015). In the present meta‐analysis, the association between sodium consumption and NAFLD risk was modified by study quality, NAFLD ascertainment methods, and sodium intake measurement tools. So, a stronger association was observed in the studies with a lower risk of bias or using estimation equations method for NAFLD diagnosis or urinary sodium excretion for exposure assessment.

Obesity, type 2 diabetes, dyslipidemia, hypertension, and metabolic syndrome are known risk factors of NAFLD (National Guideline Centre (UK), NICE Guideline, No. 49., 2016). Some previous literature supports the association between high sodium intake and these detrimental metabolic parameters (Moosavian et al., 2017; Leyvraz et al., 2018; Takase et al., 2020; Radzeviciene and Ostrauskas, 2017). A high‐salt diet boosted fat mass, plasma leptin concentration, and insulin‐stimulated glucose oxidation in the adipocytes, probably because of elevating the lipogenic capacity of white adipose tissue in rats (Fonseca‐Alaniz et al., 2007; Fonseca‐Alaniz et al., 2008). Leptin inhibits insulin secretion through several mechanisms including, suppression of glucose transport, inhibition of glucagon‐like peptide‐1‐induced expression of preproinsulin mRNA, and induction of pancreatic β‐cell apoptosis (Amitani et al., 2013). So, the potential relationship between sodium and NAFLD could partially be mediated by the effects of sodium on the NAFLD risk factors. In this regard, the association between sodium and NAFLD was attenuated and became nonsignificant after adjusting for BMI in two studies (Zhou et al., 2021; Choi et al., 2016). In another study, a strong positive correlation only was detected in overweight or obese, but not in normal‐weight participants (Emamat et al., 2021). Besides, salty foods increase thirst and promote sugar‐sweetened beverages and energy consumption (Moosavian et al., 2017). High salt intake can also represent unhealthy dietary patterns high in saturated fat, soft drinks, refined grains, and sweets, which have undeniable roles in NAFLD exacerbation (Moosavian et al., 2017; Azimi et al., 2020; Mirmiran et al., 2017). Nevertheless, there is some evidence on the independence of the mentioned association from relevant covariates (Shen et al., 2019; Huh et al., 2015).

There are few proposed mechanisms regarding the effect of sodium on NAFLD development. Uetake et al. reported aggravations of steatosis and NASH in high‐fat/high‐salt diet‐fed LOX‐1 transgenic/apoE knockout mice because of oxidative stress and inflammation induction (Uetake et al., 2015). High sodium intake also was positively related to serum C‐reactive protein and tumor necrosis factor‐α levels, respectively, in hypertensive patients and healthy adolescents (Yilmaz et al., 2012; Zhu et al., 2014). Oxidative stress and chronic inflammation play pivotal roles in the pathogenesis and progression of NAFLD (Masarone et al., 2018; Tarantino et al., 2010). A high‐salt diet also contributed to NAFLD development by the aldose reductase–fructokinase pathway activation in the liver and hypothalamus and subsequent stimulation of endogenous fructose production (Lanaspa et al., 2018). Besides, Bielinska et al. suggested an increment of plasma trimethylamine N‐oxide (TMAO) concentration and induction of gut dysbiosis in Sprague–Dawley rats following treatment with water containing sodium chloride for 2 weeks (Bielinska et al., 2018). TMAO is a metabolite of choline, betaine, and carnitine generated by gut microbiota that may aggravate steatosis by suppressing bile acid‐mediated hepatic farnesoid X receptor signaling (Tan et al., 2019). However, the mediating influence of TMAO in the effects of sodium on NAFLD development should be more investigated. Another supposed mechanism regarding the presently observed association may be the effects of a high‐salt diet on dysregulation of the renin–angiotensin system (RAS) (Emamat et al., 2021; Huh et al., 2015). Sodium loading typically inhibits renin synthesis and RAS but directly activates mineralocorticoid receptors. The RAS suppression by sodium is suggested to have weakened in diabetic and probably NAFLD patients. As a result, such patients have RAS activation that does not inhibit by a high‐salt diet. RAS and mineralocorticoid receptors activation are linked to oxidative stress and inflammation and are involved in the pathogenesis of NAFLD. Likewise, RAS inhibition may prevent NAFLD development and progression (Charytan and Forman, 2012).

Literature suggests that the sodium to potassium ratio may be a superior dietary metric than the separate sodium or potassium intakes for assessing their relation to cardiovascular disease risks (Iwahori et al., 2017). Choi et al. examined the association between sodium to potassium ratio and risk of NAFLD prevalence. They reported that those in the highest quintile of sodium and sodium to potassium ratio had a 25% and 13% higher NAFLD prevalence than ones in the lowest quintiles, respectively (Choi et al., 2016). However, in another study, comparing the highest with the lowest quartile of sodium and sodium to potassium ratio intakes resulted in 1.35 (95% CI: 1.02–1.80) and 1.53 (95% CI: 1.15–2.03) times more risk of diabetes, respectively (Hao et al., 2020). More studies are warranted to clarify the modulatory effects of potassium in such associations.

## 5 LIMITATIONS OF THE META‐ANALYSIS

The present meta‐analysis study has several limitations that should be considered during the interpretation of the findings. First, the observational design of the included investigations impeded the establishment of a causal link between salt/sodium intake and NAFLD. Second, crude OR was used in this study due to the insufficient data regarding adjusted effect sizes and differences in confounders among studies. Hence, various uncontrolled factors such as age, sex, smoking, physical activity, hemodynamic and metabolic factors, other dietary components, and unknown factors may confound the observed positive relationship between salt/sodium intake and NAFLD risk. Third, the method of NAFLD diagnosis was different between studies and was according to low accurate predictive equations in some examinations. Fourth, only one reviewed study used 24‐h urine collections for exposure measurement. Spot urine method and dietary surveys (especially dietary recalls) for salt or sodium intake assessment were utilized in other studies, which made the potential of measurement error. Fifth, the cut points of exposure classifications varied among studies that might represent different sodium consumption levels between communities. Sixth, the low number of included studies prevented the publication bias tests and a firm conclusion; thus, more research is needed to confirm the present findings. Moreover, the difference in exposure assessments prohibited dose–response analysis. Finally, the limitations resulted in very low certainty of the evidence.

## 6 CONCLUSION

In conclusion, the present meta‐analysis revealed that higher sodium intake is associated with a higher NAFLD risk. This association tended to be increased in the studies with a lower risk of bias or using the predictive equation for NAFLD ascertainment or urinary sodium excretion for a sodium intake assessment. These findings provide evidence for another health hazard of excessive salt intake and further rationale for salt restriction. It is worth mentioning that our estimated pooled effect size is unadjusted and should be interpreted with caution. Because of high heterogeneity and significant subgroup differences, further large sample and long‐term prospective cohort investigations using accurate methods are warranted to confirm the independent association between high salt intake and NAFLD risk. Besides, the effects of a low‐salt diet on NAFLD management and the potential underlying mechanisms should be investigated in future studies.

## CONFLICT OF INTEREST

The authors report no conflict of interest.

## TRANSPARENCY DECLARATION

The lead author affirms that this manuscript is an honest, accurate, and transparent account of the reported study. The reporting of this work is compliant with PRISMA guidelines. The lead author affirms that no important aspects of the study have been omitted and that any discrepancies from the study as planned (PROSPERO registration number: CRD42021232065) have been explained.

## Supporting information

Fig S1

Fig S2

Tab S1‐S4

## References

[fsn32781-bib-0001] Alferink, L. J. M. , Erler, N. S. , de Knegt, R. J. , Janssen, H. L. A. , Metselaar, H. J. , Darwish Murad, S. , & Kiefte‐de Jong, J. C. (2020). Adherence to a plant‐based, high‐fibre dietary pattern is related to regression of non‐alcoholic fatty liver disease in an elderly population. European Journal of Epidemiology, 35, 1069–1085. 10.1007/s10654-020-00627-2 32323115 PMC7695656

[fsn32781-bib-0002] Amitani, M. , Asakawa, A. , Amitani, H. , & Inui, A. (2013). The role of leptin in the control of insulin‐glucose axis. Frontiers in Neuroscience, 7, 51. 10.3389/fnins.2013.00051 23579596 PMC3619125

[fsn32781-bib-0003] Azimi, T. , Eghtesadi, S. , & Abbasi, B. (2020). The comparison of major dietary patterns in people with and without calcium oxalate kidney stone: A case‐control study. Journal of Nutrition and Food Security, 5, 365–376.

[fsn32781-bib-0004] Bansal, V. , & Mishra, S. K. (2020). Reduced‐sodium cheeses: Implications of reducing sodium chloride on cheese quality and safety. Comprehensive Reviews in Food Science and Food Safety, 19, 733–758. 10.1111/1541-4337.12524 33325171

[fsn32781-bib-0005] Baudrand, R. , Campino, C. , Carvajal, C. A. , Olivieri, O. , Guidi, G. , Faccini, G. , Vöhringer, P. A. , Cerda, J. , Owen, G. , Kalergis, A. M. , & Fardella, C. E. (2014). High sodium intake is associated with increased glucocorticoid production, insulin resistance and metabolic syndrome. Clinical Endocrinology, 80, 677–684. 10.1111/cen.12225 23594269

[fsn32781-bib-0006] Bedogni, G. , Bellentani, S. , Miglioli, L. , Masutti, F. , Passalacqua, M. , Castiglione, A. , & Tiribelli, C. (2006). The fatty liver index: A simple and accurate predictor of hepatic steatosis in the general population. BMC Gastroenterology, 6, 33. 10.1186/1471-230X-6-33 17081293 PMC1636651

[fsn32781-bib-0007] Bielinska, K. , Radkowski, M. , Grochowska, M. , Perlejewski, K. , Huc, T. , Jaworska, K. , Motooka, D. , Nakamura, S. , & Ufnal, M. (2018). High salt intake increases plasma trimethylamine N‐oxide (TMAO) concentration and produces gut dysbiosis in rats. Nutrition, 54, 33–39. 10.1016/j.nut.2018.03.004 29705499

[fsn32781-bib-0008] Birjandi, M. , Ayatollahi, S. M. T. , Pourahmad, S. , & Safarpour, A. R. (2016). Prediction and diagnosis of non‐alcoholic fatty liver disease (NAFLD) and identification of its associated factors using the classification tree method. Iranian Red Crescent Medical Journal, 18, e32858. 10.5812/ircmj.32858 28191344 PMC5292777

[fsn32781-bib-0009] Campbell, N. R. C. , Johnson, J. A. , & Campbell, T. S. (2012). Sodium consumption: An individual's choice? International Journal of Hypertension, 2012, 860954. 10.1155/2012/860954 22263106 PMC3259482

[fsn32781-bib-0010] Charytan, D. M. , & Forman, J. P. (2012). You are what you eat: Dietary salt intake and renin–angiotensin blockade in diabetic nephropathy. Kidney International, 82, 257–259. 10.1038/ki.2012.148 22791321 PMC3397395

[fsn32781-bib-0011] Chen, L.‐D. , Huang, J.‐F. , Chen, Q.‐S. , Lin, G.‐F. , Zeng, H.‐X. , Lin, X.‐F. , Lin, X.‐J. , Lin, L. I. , & Lin, Q.‐C. (2019). Validation of fatty liver index and hepatic steatosis index for screening of non‐alcoholic fatty liver disease in adults with obstructive sleep apnea hypopnea syndrome. Chinese Medical Journal, 132, 2670–2676. 10.1097/CM9.0000000000000503 31765354 PMC6940109

[fsn32781-bib-0012] Choi, Y. , Lee, J. E. , Chang, Y. , Kim, M. K. , Sung, E. , Shin, H. , & Ryu, S. (2016). Dietary sodium and potassium intake in relation to non‐alcoholic fatty liver disease. British Journal of Nutrition, 116, 1447–1456. 10.1017/S0007114516003391 27725000

[fsn32781-bib-0013] Chung, G. E. , Youn, J. , Kim, Y. S. , Lee, J. E. , Yang, S. Y. , Lim, J. H. , Song, J. H. , Doo, E. Y. , & Kim, J. S. (2019). Dietary patterns are associated with the prevalence of nonalcoholic fatty liver disease in Korean adults. Nutrition, 62, 32–38. 10.1016/j.nut.2018.11.021 30826597

[fsn32781-bib-0014] D’Elia, L. , Rossi, G. , Ippolito, R. , Cappuccio, F. P. , & Strazzullo, P. (2012). Habitual salt intake and risk of gastric cancer: A meta‐analysis of prospective studies. Clinical Nutrition, 31, 489–498. 10.1016/j.clnu.2012.01.003 22296873

[fsn32781-bib-0015] Dai, W. , Ye, L. , Liu, A. , Wen, S. W. , Deng, J. , Wu, X. , & Lai, Z. (2017). Prevalence of nonalcoholic fatty liver disease in patients with type 2 diabetes mellitus: A meta‐analysis. Medicine, 96, e8179. 10.1097/MD.0000000000008179 28953675 PMC5626318

[fsn32781-bib-0016] Defagó, M. , & Perovic, N. (2015). Nutritional epidemiological tools for sodium intake. Journal of Hypertension (Los Angel), 4,1–3. 10.4172/2167-1095.1000208

[fsn32781-bib-0017] Dongiovanni, P. , Anstee, Q. M. , & Valenti, L. (2013). Genetic predisposition in NAFLD and NASH: Impact on severity of liver disease and response to treatment. Current Pharmaceutical Design, 19, 5219–5238. 23394097 10.2174/13816128113199990381 PMC3850262

[fsn32781-bib-0018] Emamat, H. , Farhadnejad, H. , Movahedian, M. , Tangestani, H. , Mirmiran, P. , & Hekmatdoost, A. (2021). Dietary sodium intake in relation to non‐alcoholic fatty liver disease risk: A case‐control study. Nutrition & Food Science, 51(3), 541–550. 10.1108/NFS-05-2020-0183

[fsn32781-bib-0019] Fonseca‐Alaniz, M. H. , Brito, L. C. , Borges‐Silva, C. N. , Takada, J. , Andreotti, S. , & Lima, F. B. (2007). High dietary sodium intake increases white adipose tissue mass and plasma leptin in rats. Obesity (Silver Spring), 15, 2200–2208. 10.1038/oby.2007.261 17890487

[fsn32781-bib-0020] Fonseca‐Alaniz, M. H. , Takada, J. , Andreotti, S. , De Campos, T. B. F. , Campaña, A. B. , Borges‐Silva, C. N. , & Lima, F. B. (2008). High sodium intake enhances insulin‐stimulated glucose uptake in rat epididymal adipose tissue. Obesity (Silver Spring), 16, 1186–1192. 10.1038/oby.2008.69 18369340

[fsn32781-bib-0021] Guyatt, G. H. , Oxman, A. D. , Kunz, R. , Brozek, J. , Alonso‐Coello, P. , Rind, D. , Devereaux, P. J. , Montori, V. M. , Freyschuss, B. O. , Vist, G. , Jaeschke, R. , Williams, J. W. , Murad, M. H. , Sinclair, D. , Falck‐Ytter, Y. , Meerpohl, J. , Whittington, C. , Thorlund, K. , Andrews, J. , & Schünemann, H. J. (2011). GRADE guidelines 6. Rating the quality of evidence—imprecision. Journal of Clinical Epidemiology, 64, 1283–1293. 10.1016/j.jclinepi.2011.01.012 21839614

[fsn32781-bib-0022] Guyatt, G. H. , Oxman, A. D. , Kunz, R. , Woodcock, J. , Brozek, J. , Helfand, M. , Alonso‐Coello, P. , Falck‐Ytter, Y. , Jaeschke, R. , Vist, G. , Akl, E. A. , Post, P. N. , Norris, S. , Meerpohl, J. , Shukla, V. K. , Nasser, M. , & Schünemann, H. J. (2011). GRADE guidelines: 8. Rating the quality of evidence—indirectness. Journal of Clinical Epidemiology, 64, 1303–1310. 10.1016/j.jclinepi.2011.04.014 21802903

[fsn32781-bib-0023] Guyatt, G. H. , Oxman, A. D. , Kunz, R. , Woodcock, J. , Brozek, J. , Helfand, M. , Alonso‐Coello, P. , Glasziou, P. , Jaeschke, R. , Akl, E. A. , Norris, S. , Vist, G. , Dahm, P. , Shukla, V. K. , Higgins, J. , Falck‐Ytter, Y. , & Schünemann, H. J. (2011). GRADE guidelines: 7. Rating the quality of evidence—inconsistency. Journal of Clinical Epidemiology, 64, 1294–1302. 10.1016/j.jclinepi.2011.03.017 21803546

[fsn32781-bib-0024] Guyatt, G. H. , Oxman, A. D. , Vist, G. , Kunz, R. , Brozek, J. , Alonso‐Coello, P. , Montori, V. , Akl, E. A. , Djulbegovic, B. , & Falck‐Ytter, Y. (2011). GRADE guidelines: 4. Rating the quality of evidence—study limitations (risk of bias). Journal of Clinical Epidemiology, 64, 407–415. 10.1016/j.jclinepi.2010.07.017 21247734

[fsn32781-bib-0025] Han, S. , Cheng, D. , Liu, N. , & Kuang, H. (2018). The relationship between diabetic risk factors, diabetic complications and salt intake. Journal of Diabetes and Its Complications, 32, 531–537. 10.1016/j.jdiacomp.2018.02.003 29534865

[fsn32781-bib-0026] Hao, G. , Liu, K. , Halbert, J. D. , Chen, H. , Wu, J. , & Jing, C. (2020). Dietary sodium and potassium and risk of diabetes: A prospective study using data from the China health and nutrition survey. Diabetes & Metabolism, 46, 377–383. 10.1016/j.diabet.2019.12.002 31838058

[fsn32781-bib-0027] Huh, J. H. , Lee, K. J. , Lim, J. S. , Lee, M. Y. , Park, H. J. , Kim, M. Y. , Kim, J. W. , Chung, C. H. , Shin, J. Y. , Kim, H.‐S. , Kwon, S. O. , & Baik, S. K. (2015). High dietary sodium intake assessed by estimated 24‐h urinary sodium excretion is associated with NAFLD and hepatic fibrosis. PLoS One, 10, e0143222. 10.1371/journal.pone.0143222 26571018 PMC4646649

[fsn32781-bib-0028] Iwahori, T. , Miura, K. , & Ueshima, H. (2017). Time to consider use of the sodium‐to‐potassium ratio for practical sodium reduction and potassium increase. Nutrients, 9, 700. 10.3390/nu9070700 PMC5537815 28678188

[fsn32781-bib-0029] Kneeman, J. M. , Misdraji, J. , & Corey, K. E. (2012). Secondary causes of nonalcoholic fatty liver disease. Therapeutic Advances in Gastroenterology, 5, 199–207. 10.1177/1756283X11430859 22570680 PMC3342568

[fsn32781-bib-0030] Lanaspa, M. A. , Kuwabara, M. , Andres‐Hernando, A. , Li, N. , Cicerchi, C. , Jensen, T. , Orlicky, D. J. , Roncal‐Jimenez, C. A. , Ishimoto, T. , Nakagawa, T. , Rodriguez‐Iturbe, B. , MacLean, P. S. , & Johnson, R. J. (2018). High salt intake causes leptin resistance and obesity in mice by stimulating endogenous fructose production and metabolism. Proceedings of the National Academy of Sciences USA, 115, 3138–3143. 10.1073/pnas.1713837115 PMC5866545 29507217

[fsn32781-bib-0031] Lee, J.‐H. , Kim, D. , Kim, H. J. , Lee, C.‐H. , Yang, J. I. , Kim, W. , Kim, Y. J. , Yoon, J.‐H. , Cho, S.‐H. , Sung, M.‐W. , & Lee, H.‐S. (2010). Hepatic steatosis index: A simple screening tool reflecting nonalcoholic fatty liver disease. Digestive and Liver Disease: Official Journal of the Italian Society of Gastroenterology and the Italian Association for the Study of the Liver, 42, 503–508. 10.1016/j.dld.2009.08.002 19766548

[fsn32781-bib-0032] Leyvraz, M. , Chatelan, A. , da Costa, B. R. , Taffé, P. , Paradis, G. , Bovet, P. , Bochud, M. , & Chiolero, A. (2018). Sodium intake and blood pressure in children and adolescents: A systematic review and meta‐analysis of experimental and observational studies. International Journal of Epidemiology, 47, 1796–1810. 10.1093/ije/dyy121 29955869

[fsn32781-bib-0033] Liberati, A. , Altman, D. G. , Tetzlaff, J. , Mulrow, C. , Gøtzsche, P. C. , Ioannidis, J. P. , Clarke, M. , Devereaux, P. J. , Kleijnen, J. , & Moher, D. (2009). The PRISMA statement for reporting systematic reviews and meta‐analyses of studies that evaluate health care interventions: Explanation and elaboration. Journal of Clinical Epidemiology, 62, e1–e34. 19631507 10.1016/j.jclinepi.2009.06.006

[fsn32781-bib-0034] Masarone, M. , Rosato, V. , Dallio, M. , Gravina, A. G. , Aglitti, A. , Loguercio, C. , Federico, A. , & Persico, M. (2018). Role of oxidative stress in pathophysiology of nonalcoholic fatty liver disease. Oxidative Medicine and Cellular Longevity, 2018, 9547613. 10.1155/2018/9547613 29991976 PMC6016172

[fsn32781-bib-0035] Menyanu, E. , Russell, J. , & Charlton, K. (2019). Dietary sources of salt in low‐ and middle‐income countries: A systematic literature review. International Journal of Environmental Research and Public Health, 16, 2082. 10.3390/ijerph16122082 PMC6617282 31212868

[fsn32781-bib-0036] Mirmiran, P. , Amirhamidi, Z. , Ejtahed, H.‐S. , Bahadoran, Z. , & Azizi, F. (2017). Relationship between diet and non‐alcoholic fatty liver disease: A review article. Iranian Journal of Public Health, 46, 1007. 28894701 PMC5575379

[fsn32781-bib-0037] Modesti, P. A. , Reboldi, G. , Cappuccio, F. P. , Agyemang, C. , Remuzzi, G. , Rapi, S. , Perruolo, E. , & Parati, G. (2016). Panethnic differences in blood pressure in Europe: A systematic review and meta‐analysis. PLoS One, 11, e0147601. 10.1371/journal.pone.0147601 26808317 PMC4725677

[fsn32781-bib-0038] Moosavian, S. P. , Haghighatdoost, F. , Surkan, P. J. , & Azadbakht, L. (2017). Salt and obesity: A systematic review and meta‐analysis of observational studies. International Journal of Food Sciences and Nutrition, 68, 265–277. 10.1080/09637486.2016.1239700 27706955

[fsn32781-bib-0039] Nassir, F. , Rector, R. S. , Hammoud, G. M. , Ibdah, J. A. (2015). Pathogenesis and prevention of hepatic steatosis. Gastroenterology & Hepatology, 11, 167. 27099587 PMC4836586

[fsn32781-bib-0040] National Guideline Centre (UK), NICE Guideline, No. 49. (2016). Non‐Alcoholic Fatty Liver Disease: Assessment and Management. 27441333

[fsn32781-bib-0041] Nivukoski, U. , Niemelä, M. , Bloigu, A. , Bloigu, R. , Aalto, M. , Laatikainen, T. , & Niemelä, O. (2020). Combined effects of lifestyle risk factors on fatty liver index. BMC Gastroenterology, 20, 1–10. 10.1186/s12876-020-01270-7 PMC7157978 32293287

[fsn32781-bib-0042] Portela, C. L. M. , Sampaio, H. A. C. , de Melo, M. L. P. , Ferreira Carioca, A. A. , Maia Pinto, F. J. , & Machado Arruda, S. P. (2015). Nutritional status, diet and non‐alcoholic fatty liver disease in elders. Nutricion Hospitalaria, 32, 2038–2045. 26545658 10.3305/nh.2015.32.5.9674

[fsn32781-bib-0043] Powles, J. , Fahimi, S. , Micha, R. , Khatibzadeh, S. , Shi, P. , Ezzati, M. , Engell, R. E. , Lim, S. S. , Danaei, G. , & Mozaffarian, D. , Global Burden of Diseases Nutrition and Chronic Diseases Expert Group (NutriCoDE) (2013). Global, regional and national sodium intakes in 1990 and 2010: A systematic analysis of 24 h urinary sodium excretion and dietary surveys worldwide. British Medical Journal Open, 3, e003733. 10.1136/bmjopen-2013-003733 PMC3884590 24366578

[fsn32781-bib-0044] Radzeviciene, L. , & Ostrauskas, R. (2017). Adding salt to meals as a risk factor of type 2 diabetes mellitus: A case–control study. Nutrients, 9, 67. 10.3390/nu9010067 PMC5295111 28098780

[fsn32781-bib-0045] Schünemann, H. J. , Cuello, C. , Akl, E. A. , Mustafa, R. A. , Meerpohl, J. J. , Thayer, K. , Morgan, R. L. , Gartlehner, G. , Kunz, R. , Katikireddi, S. V. , Sterne, J. , Higgins, J. P. T. , & Guyatt, G. (2019). GRADE guidelines: 18. How ROBINS‐I and other tools to assess risk of bias in nonrandomized studies should be used to rate the certainty of a body of evidence. Journal of Clinical Epidemiology, 111, 105–114. 10.1016/j.jclinepi.2018.01.012 29432858 PMC6692166

[fsn32781-bib-0046] Shen, X. , Jin, C. , Wu, Y. , Zhang, Y. , Wang, X. , Huang, W. , Li, J. , Wu, S. , & Gao, X. (2019). Prospective study of perceived dietary salt intake and the risk of non‐alcoholic fatty liver disease. Journal of Human Nutrition & Dietetics, 32, 802–809. 10.1111/jhn.12674 31209928

[fsn32781-bib-0047] Strazzullo, P. , D'Elia, L. , Kandala, N.‐B. , & Cappuccio, F. P. (2009). Salt intake, stroke, and cardiovascular disease: Meta‐analysis of prospective studies. BMJ, 339, b4567. 10.1136/bmj.b4567 19934192 PMC2782060

[fsn32781-bib-0048] Subasinghe, A. K. , Arabshahi, S. , Busingye, D. , Evans, R. G. , Walker, K. Z. , Riddell, M. A. , & Thrift, A. G. (2015). Association between salt and hypertension in rural and urban populations of low to middle income countries: A systematic review and meta‐analysis of population based studies. Asia Pacific Journal of Clinical Nutrition, 25, 402–413. 10.6133/apjcn.2016.25.2.25 27222425

[fsn32781-bib-0049] Takase, H. , Machii, M. , Nonaka, D. , Ohno, K. , Takayama, S. , Sugiura, T. , Ohte, N. , & Dohi, Y. (2020). Excessive salt intake is a significant predictor for future development of metabolic syndrome in the general population. European Heart Journal, 41, ehaa946. 3058. 10.1093/ehjci/ehaa946.3058

[fsn32781-bib-0050] Tan, X. , Liu, Y. , Long, J. , Chen, S. , Liao, G. , Wu, S. , Li, C. , Wang, L. , Ling, W. , & Zhu, H. (2019). Trimethylamine N‐oxide aggravates liver steatosis through modulation of bile acid metabolism and inhibition of farnesoid x receptor signaling in nonalcoholic fatty liver disease. Molecular Nutrition & Food Research, 63, 1900257. 10.1002/mnfr.201900257 31095863

[fsn32781-bib-0051] Tarantino, G. , Savastano, S. , & Colao, A. (2010). Hepatic steatosis, low‐grade chronic inflammation and hormone/growth factor/adipokine imbalance. World Journal of Gastroenterology, 16, 4773–4783. 10.3748/wjg.v16.i38.4773 20939105 PMC2955246

[fsn32781-bib-0052] Tarsilla, M. (2010). Cochrane handbook for systematic reviews of interventions. Journal of Multidisciplinary Evaluation, 6, 142–148.

[fsn32781-bib-0053] Uetake, Y. , Ikeda, H. , Irie, R. , Tejima, K. , Matsui, H. , Ogura, S. , Wang, H. , Mu, S. Y. , Hirohama, D. , Ando, K. , Sawamura, T. , Yatomi, Y. , Fujita, T. , & Shimosawa, T. (2015). High‐salt in addition to high‐fat diet may enhance inflammation and fibrosis in liver steatosis induced by oxidative stress and dyslipidemia in mice. Lipids in Health and Disease, 14, 1–8. 10.1186/s12944-015-0002-9 25888871 PMC4337194

[fsn32781-bib-0054] United States Department of Agriculture Aimapp, [internet] (2015), Retrieved from https://www.fsis.usda.gov/wps/portal/fsis/topics/food‐safety‐education/get‐answers/food‐safety‐fact‐sheets/food‐labeling/additives‐in‐meat‐and‐poultry‐products/additives‐in‐meat‐and‐poultry‐products

[fsn32781-bib-0055] van den Berg, E. H. , Gruppen, E. G. , Blokzijl, H. , Bakker, S. J. L. , & Dullaart, R. P. F. (2019). Higher sodium intake assessed by 24 hour urinary sodium excretion is associated with non‐alcoholic fatty liver disease: The PREVEND cohort study. Journal of Clinical Medicine, 8, 2157. 10.3390/jcm8122157 PMC6947413 31817623

[fsn32781-bib-0056] Wells, G. A. , Shea, B. , O’ Connell, D. , Peterson, J. , Welch, V. , Losos, M. , & Tugwell, P. (2000). The Newcastle‐Ottawa Scale (NOS) for assessing the quality of nonrandomised studies in meta‐analyses: Oxford.

[fsn32781-bib-0057] Wong, C. R. , & Lim, J. K. (2018). The association between nonalcoholic fatty liver disease and cardiovascular disease outcomes. Clinics in Liver Disease, 12, 39. 10.1002/cld.721 PMC6385911 30988909

[fsn32781-bib-0058] World Health Organization (WHO) Sr, [internet] (2020). Retrieved from https://www.who.int/news‐room/fact‐sheets/detail/salt‐reduction#:~:text=For%20adults%3A%20WHO%20recommends%20that,relative%20to%20those%20of%20adults

[fsn32781-bib-0059] Yang, C.‐Q. , Shu, L. , Wang, S. , Wang, J.‐J. , Zhou, Y. U. , Xuan, Y.‐J. , & Wang, S.‐F. (2015). Dietary patterns modulate the risk of non‐alcoholic fatty liver disease in Chinese adults. Nutrients, 7, 4778–4791. 10.3390/nu7064778 26083112 PMC4488813

[fsn32781-bib-0060] Yari, Z. , Cheraghpour, M. , Aghamohammadi, V. , Alipour, M. , Ghanei, N. , & Hekmatdoost, A. (2020). Energy‐dense nutrient‐poor snacks and risk of non‐alcoholic fattyliver disease: A case‐control study in Iran. BMC Research Notes, 13, 221. 32299509 10.1186/s13104-020-05063-9 PMC7164180

[fsn32781-bib-0061] Yilmaz, R. , Akoglu, H. , Altun, B. , Yildirim, T. , Arici, M. , & Erdem, Y. (2012). Dietary salt intake is related to inflammation and albuminuria in primary hypertensive patients. European Journal of Clinical Nutrition, 66, 1214–1218. 10.1038/ejcn.2012.110 22909578

[fsn32781-bib-0062] Younossi, Z. M. , Koenig, A. B. , Abdelatif, D. , Fazel, Y. , Henry, L. , & Wymer, M. (2016). Global epidemiology of nonalcoholic fatty liver disease—meta‐analytic assessment of prevalence, incidence, and outcomes. Hepatology, 64, 73–84. 10.1002/hep.28431 26707365

[fsn32781-bib-0063] Zhou, L. , Tian, Y. U. , Fu, J.‐J. , Jiang, Y.‐Y. , Bai, Y.‐M. , Zhang, Z.‐H. , Hu, X.‐H. , Lian, H.‐W. , Guo, M. , Yang, Z.‐X. , & Zhao, L.‐C. (2017). Validation of spot urine in predicting 24‐h sodium excretion at the individual level. American Journal of Clinical Nutrition, 105, 1291–1296. 10.3945/ajcn.116.147553 28356277

[fsn32781-bib-0064] Zhou, L. , Yang, Y. , Feng, Y. , Zhao, X. , Fan, Y. , Rong, J. , Zhao, L. , & Yu, Y. (2021). Association between dietary sodium intake and non‐alcoholic fatty liver disease in the US population. Public Health Nutrition, 24, 993–1000. 10.1017/S136898001900483X 32312347 PMC10195540

[fsn32781-bib-0065] Zhu, H. , Pollock, N. K. , Kotak, I. , Gutin, B. , Wang, X. , Bhagatwala, J. , Parikh, S. , Harshfield, G. A. , & Dong, Y. (2014). Dietary sodium, adiposity, and inflammation in healthy adolescents. Pediatrics, 133, e635–e642. 10.1542/peds.2013-1794 24488738 PMC3934330

