# Supplementary figures and images for: Sodium in relation with nonalcoholic fatty liver disease: A systematic review and meta‐analysis of observational studies
Source: Food Sci Nutr. 2022 Feb 15;10(5):1579–91. doi: 10.1002/fsn3.2781 (PMC9094449; doi:10.1002/fsn3.2781)

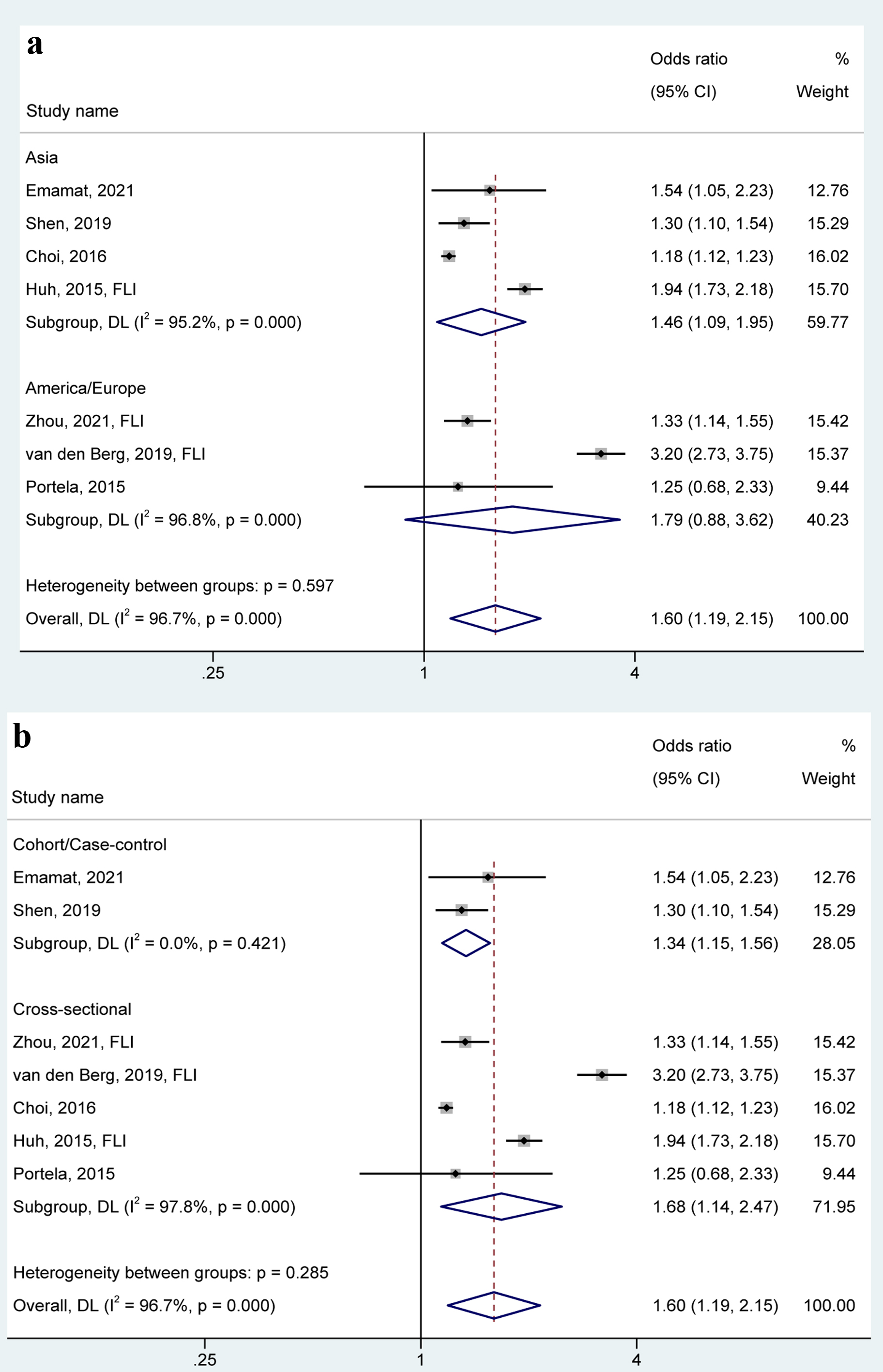

Supplement: Supplementary file 1 — Fig S1 [file FSN3-10-1579-s003.jpg]

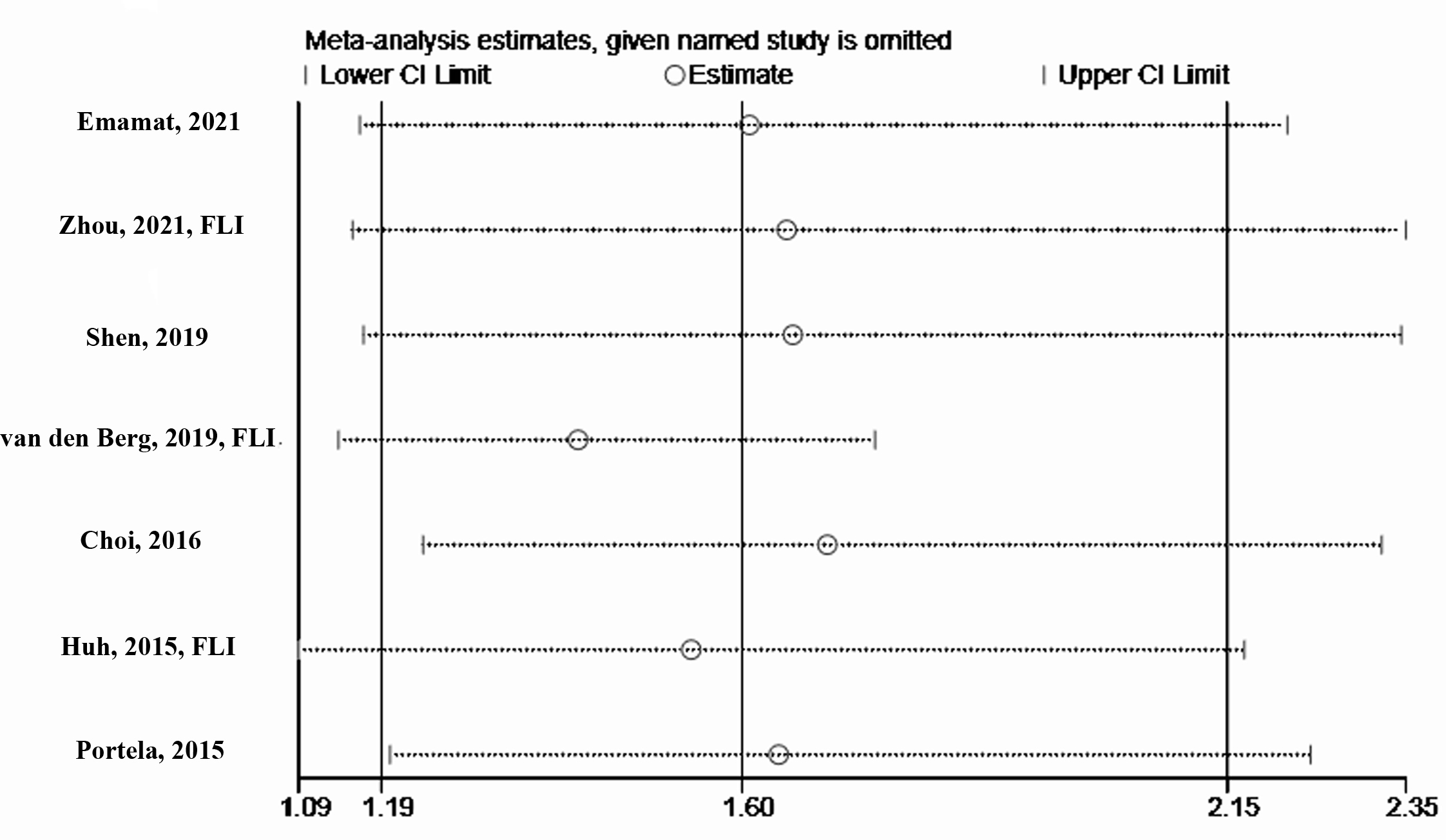

Supplement: Supplementary file 2 — Fig S2 [file FSN3-10-1579-s002.jpg]
