# Supplementary material for: Sodium in relation with nonalcoholic fatty liver disease: A systematic review and meta‐analysis of observational studies
Source: Food Sci Nutr. 2022 Feb 15;10(5):1579–91. doi: 10.1002/fsn3.2781 (PMC9094449; doi:10.1002/fsn3.2781)
Supplement: Supplementary file 3 — Tab S1‐S4 [file FSN3-10-1579-s001.docx]

**Sodium in relation with non-alcoholic fatty liver disease: A systematic review and meta-analysis of observational studies**

**Table S1: PubMed Search strategy**

| **Search strategy** | **Number needed to read (NNR)** |
| --- | --- |
| (fatty liver[MeSH Terms] OR Non-alcoholic Fatty Liver Disease[MeSH Terms] OR "fatty liver" [tiab] OR "nonalcoholic fatty liver disease"[tiab] OR "non-alcoholic fatty liver"[tiab] OR "non alcoholic Fatty Liver"[tiab] OR steato*[tiab] OR nafld[tiab] OR nafl[tiab] OR nash[tiab] OR "nonalcoholic steatohepatitis"[tiab] OR "non-alcoholic steatohepatitis"[tiab]) AND ((Sodium, Dietary[MeSH Terms] OR Hyponatremia[MeSH Terms] OR Hypernatremia[MeSH Terms] OR Hyponatremia[tiab] OR Hypernatremia[tiab] OR dysnatremia[tiab]) OR ((Sodium Chloride[MeSH Terms] OR salt[all] OR sodium[all]) AND (diet[MeSH Terms] OR diet*[tiab] OR intake*[tiab] OR consum*[tiab] OR restrict*[tiab] OR reduc*[tiab]))) | 20 |

**Table S2: List of excluded studies following full-text assessment**

| **No.** | **Title of article** | **Reason of exclusion** |
| --- | --- | --- |
| 1 | Association of the dietary patterns with the risk of non-alcoholic fatty liver disease among Iranian population: a case-control study | Dietary patterns |
| 2 | Association between nutrient patterns and fatty liver index: Baseline survey of the Japan Multi-Institutional Collaborative Cohort Study in Tokushima, Japan | Dietary patterns |
| 3 | Dietary patterns are associated with the prevalence of nonalcoholic fatty liver disease in Korean adults | Dietary patterns |
| 4 | Dietary patterns and risk of non-alcoholic fatty liver disease | Dietary patterns |
| 5 | Dietary patterns modulate the risk of non-alcoholic fatty liver disease in Chinese adults | Dietary patterns |
| 6 | Dietary Factors in Relation to Liver Fat Content: A Cross-sectional Study | Dietary patterns |
| 7 | Elevated serum levels of aminotransferases in relation to unhealthy foods intake: Tehran lipid and glucose study | Salty foods |
| 8 | Energy-dense nutrient-poor snacks and risk of non-alcoholic fattyliver disease: A case-control study in Iran | Salty foods |
| 9 | Associations Among Methylene Tetrahydrofolate Reductase rs1801133 C677T Gene Variant, Food Groups, and Non-alcoholic Fatty Liver Disease Risk in the Chinese Population | Salty foods |
| 10 | A cross-sectional study assessing dietary intake and physical activity in canadian patients with nonalcoholic fatty liver disease vs healthy controls | On the amount of sodium intake in fatty liver patients |
| 11 | Nutritional assessments of patients with non-alcoholic fatty liver disease | On the amount of sodium intake in fatty liver patients |
| 12 | Analysis of the dietary factors associated with suspected pediatric nonalcoholic fatty liver disease and potential liver fibrosis: Korean National Health and Nutrition Examination Survey 2014-2017 | On the amount of sodium intake in fatty liver patients |
| 13 | Assessment of Diet and Physical Activity in Paediatric Non-Alcoholic Fatty Liver Disease Patients: A United Kingdom Case Control Study | On the amount of sodium intake in fatty liver patients |
| 14 | Associations between intakes of individual nutrients or whole food groups and non-alcoholic fatty liver disease among Korean adults | On the amount of sodium intake in fatty liver patients |
| 15 | Cross-sectional analysis of the health profile and dietary intake of a sample of canadian adults diagnosed with non-alcoholic fatty liver disease | On the amount of sodium intake in fatty liver patients |
| 16 | Associations between Dietary Nutrient Intakes and Hepatic Lipid Contents in NAFLD Patients Quantified by 1H-MRS and Dual-Echo MRI | Other dietary factors |
| 17 | Improved Diet Quality Associates With Reduction in Liver Fat, Particularly in Individuals With High Genetic Risk Scores for Nonalcoholic Fatty Liver Disease | Other dietary factors |
| 18 | Association of Genetic and Environmental Factors with Non-Alcoholic Fatty Liver Disease in a Chinese Han Population | Other dietary factors |
| 19 | Effects of Some Food Components on Non-Alcoholic Fatty Liver Disease Severity: Results from a Cross-Sectional Study | Other dietary factors |
| 20 | Non-alcoholic fatty liver disease in overweight children: Role of fructose intake and dietary pattern | Other dietary factors |
| 21 | Association between Different Animal Protein Sources and Liver Status in Obese Subjects with Non-Alcoholic Fatty Liver Disease: Fatty Liver in Obesity (FLiO) Study | Other dietary factors |
| 22 | Associations between dietary factors and markers of NAFLD in a general Dutch adult population | Other dietary factors |
| 23 | High salt intake causes leptin resistance and obesity in mice by stimulating endogenous fructose production and metabolism | Not reported sufficient data |
| 24 | Assessment of Lifestyle Factors Helps to Identify Liver Fibrosis Due to Non-Alcoholic Fatty Liver Disease in Obesity | Hepatic fibrosis |

**Table S3: Certainty of evidence according to GRADE approach of association between salt/sodium intake and NAFLD**

| **Certainty assessment** | | | | | | | **No of patients** | | **Effect** | | **Certainty** | **Importance** |
| --- | --- | --- | --- | --- | --- | --- | --- | --- | --- | --- | --- | --- |
| **No of studies** | **Study design** | **Risk of bias** | **Inconsistency** | **Indirectness** | **Imprecision** | **Other considerations** | **[Participants]** | **[Cases]** | **Relative (95% CI)** | **Absolute (95% CI)** |  |  |
| **Risk of fatty liver** | | | | | | | | | | | | |
| 7 | observational studies (mostly cross-sectional) | serious^a^ | not serious^b^ | very serious^c^ | Serious^d^ | none | 162550 | 33242 (20.5%) | **OR 1.60** (1.19 to 2.15) | **87 more per 1,000** (from 30 more to 151 more) | ⨁◯◯◯ Very low | CRITICAL |

**GRADE:** Grading of Recommendations Assessment, Development, and Evaluation; **CI:** confidence interval; **OR:** odds ratio

Explanations:

a. Serious risk of bias since most of the included studies had high risk of bias for "Ascertainment of exposure".

b. Very serious inconsistency since I^2^ was >90%. However, I^2^ was<50% in the subgroup of Dietary instruments (Exposure), and the pooled estimate was almost similar to the main analysis (OR=1.23, 95%CI= 1.15 to 1.32). Not downgraded.

c. Very serious indirectness since most of the studies were done in Asia and assessed the sodium intakes by methods other than a 24-h urinary collection (as the gold standard). Downgraded.

d. Serious imprecision since the 95% CI (1.19, 2.15) was lower than the minimally important difference for clinical relevance (1.25).

**Fig.S1:** Forest plot (random-effects model) depicting the association of salt/sodium intake (highest vs. lowest category) and risk of non-alcoholic fatty liver disease sub-grouped by (a) Region, and (b) Study design

**Fig.S2:** Sensitivity analysis
